# Searching for Strep A in the clinical environment during a human challenge trial: a sub-study protocol

**DOI:** 10.1099/acmi.0.000650.v3

**Published:** 2023-09-20

**Authors:** Stephanie L. Enkel, Thel K. Hla, Bernadette Wong, Janessa Pickering, Timothy C. Barnett, Hannah M. M. Thomas, Nina Lansbury, Jonathan R. Carapetis, Laurens Manning, Asha C. Bowen

**Affiliations:** ^1^​ Wesfarmers Centre of Vaccines and Infectious Diseases, Telethon Kids Institute, University of Western Australia, Nedlands, WA, Australia; ^2^​ Medical School, University of Western Australia, Crawley, WA, Australia; ^3^​ Department of Infectious Diseases, Fiona Stanley Hospital, Murdoch, WA, Australia; ^4^​ Marshall Centre for Infectious Diseases Research and Training, School of Biomedical Sciences, University of Western Australia, Nedlands, WA, Australia; ^5^​ School of Public Health, University of Queensland, Brisbane, QLD, Australia; ^6^​ Department of Infectious Diseases, Perth Children’s Hospital, Nedlands, WA, Australia; ^7^​ Menzies School of Health Research, Darwin, NT, Australia

**Keywords:** *Streptococcus pyogenes*, transmission, epidemiology, infection

## Abstract

*
Streptococcus pyogenes* (also known as group A *Streptococcus *, Strep A) is an obligate human pathogen with significant global morbidity and mortality. Transmission is believed to occur primarily between individuals via respiratory droplets, but knowledge about other potential sources of transmission via aerosols or the environment is limited. Such knowledge is required to design optimal interventions to control transmission, particularly in endemic settings. We aim to detail an experimental methodology to assess the transmission potential of Strep A in a clinical environment. We will examine potential sources of transmission in up to 20 participants recruited to the Controlled human infection for penicillin against *Streptococcus pyogenes* (CHIPS) Trial. Three approaches to understanding transmission will be used: the use of selective agar settle plates to capture possible droplet or airborne spread of Strep A; measurement of the possible distance of Strep A droplet spread during conversation; and environmental swabbing of personal and common high-touch items to detect the presence of Strep A on hard and soft surfaces. All methods are designed to allow for an assessment of transmission potential by symptomatic, asymptomatic and non-cases. Ethical approval has been obtained through Bellberry Human Research Ethics Committee (approval 2021-03-295). Trial registration number: ACTRN12621000751875. Any results elicited from these experiments will be of benefit to the scientific literature in improving our knowledge of opportunities to prevent Strep A transmission as a direct component of the primordial prevention of rheumatic fever. Findings will be reported at local, national and international conferences and in peer-reviewed journals.

## Data Summary

No data were generated or reused in the research.

**Impact Statement**

This proposed investigation provides a nascent opportunity to conduct experimentation regarding the transmissibility of Strep A bacteria, a pathogen causing significant global infection, illness and death. To date, questions remain as to how Strep A can spread from person to person, with emerging research implicating routes such as airborne and small droplet. We outline three experiments occurring alongside the Controlled human infection for penicillin against *Streptococcus pyogenes* (CHIPS) Trial whereby participants will be purposely challenged with Strep A and given different doses of penicillin, with the aim of determining the minimum dose required to prevent the development of Strep A pharyngitis. Any results obtained from these experiments will be of benefit to the scientific literature in improving our knowledge of Strep A prevention opportunities.

## Introduction

*Streptococcus pyogenes* (Group A *Streptococcus *, Strep A) is an obligate human pathogen with no known animal or environmental reservoir [1]. Strep A infections present with diverse clinical phenotypes, including superficial (i.e., pharyngitis, impetigo) and invasive (i.e., bacteraemia, necrotizing fasciitis) infections [2, 3]. Globally, Strep A is estimated to cause over 162 million cases of impetigo (skin sores) at any one time, 616 million cases of acute pharyngitis (sore throat) per year and 177, 000 deaths due to invasive disease [4]. This burden is exacerbated by the potential for Strep A infection to cause delayed, immune-mediated conditions, such as acute rheumatic fever (ARF) and rheumatic heart disease (RHD), which constitute significant morbidity and mortality even with treatment [5]. Estimates suggest 471, 000 cases of ARF annually, with 40 million people presently affected by RHD and 340,000 annual deaths [6]. This array of clinical manifestations places Strep A in the top 10 most prevalent pathogens globally [3, 7, 8].

Although most cases of pharyngitis are caused by respiratory viruses [9], Strep A is the primary cause of bacterial pharyngitis, isolated in 10–40 % of cases in children [10]. Colloquially known as ‘Strep throat’, symptoms include pain when swallowing, a temperature over 38 °C, swollen tonsils and tonsillar or pharyngeal exudates [11]. To date, Strep A remains reliably sensitive to penicillin, which is the cornerstone of treatment [12]. First-line treatment is oral penicillin therapy for 10 days or one injection of intramuscular benzathine penicillin [11]. Given the lower rate of bacterial pharyngitis compared with viral pharyngitis and resolution of symptoms for most pharyngitis without treatment, prescription of antibiotics without confirmation of Strep A by throat swab is only recommended for those at high risk of Strep A immune-mediated diseases [13]. In Australia, this includes Aboriginal and Torres Strait Islander people, who are at high risk of ARF and RHD [13].

Existing evidence points to the transmission of Strep A primarily by large respiratory droplets [14–16]. Contemporary methods have worked to verify this and through the use of biological swabs, environmental swabs and environmental settle plates have suggested other mechanisms, including small droplet (nasal secretions, sputum or spit) [17, 18], skin-to-skin contact [19, 20], direct contact with bedding, fabrics and surfaces [21, 22], and via food [23, 24] and insects [25–27]. A study completed in the United Kingdom (UK) during a recent scarlet fever outbreak suggested the potential for Strep A to be disseminated via the airborne route, as measured by the placement of settle plates at various heights above the droplet-generating potential of small children [28]. Research in clinical settings has also used settle plates to identify transmission during outbreaks of Strep A causing invasive disease [29]. While verification of many of these transmission mechanisms is still required, it is acknowledged that the type of spread from infected to uninfected individuals may vary according to the clinical manifestation of infection [30].

This protocol, embedded in a human challenge study evaluating the minimum concentration of penicillin required to prevent pharyngitis infection [31], defines the experimental methodology we designed to further understand the transmission potential of Strep A pharyngitis in a controlled setting.

## Method and analysis

### Study design

This sub-study forms a component of the Controlled human infection for penicillin against *Streptococcus pyogenes* (CHIPS) Trial, (registration number ACTRN12621000751875), a double-blind, placebo-controlled, randomized trial using a previously described human challenge model [31]. Briefly, the CHIPS Trial is designed to determine the optimal dose of penicillin needed to prevent Strep A pharyngitis, conducted in a purpose-built research facility resembling a hospital ward managed by a contract research organization (CRO). All potential participants undertake screening throat swabs and a serum *emm*75 type-specific serology to exclude Strep A carriage or prior infection with the same strain. Each participant is then randomized to receive one of five doses of steady-state penicillin infusions – 0 (placebo), 3, 6, 12 and 20 ng/mL – prior to receiving a direct oropharyngeal challenge with an inoculum of the *emm*75 strain of Strep A via a ‘reverse’ throat swab [32, 33]. Participants are then monitored for development of Strep A pharyngitis according to pre-specified outcome criteria during a confinement period lasting up to 6 days. All participants will be treated as possible infections. This sub-study involves three separate – but related – experiments (detailed below).

### Study objectives

The primary objective of this sub-study within CHIPS is to assess the transmission potential of Strep A in a clinical environment, where the timing and infective dose of Strep A causing potential pharyngitis are pre-defined. Secondary objectives include determining the distance of Strep A droplet spread during conversation, investigating potential airborne spread of Strep A, and the isolation of Strep A from hard and soft surfaces in the clinical environment. It is also anticipated that this sub-study may allow for determination of the impact of different doses of treatment with penicillin against the transmission potential of Strep A.

### Sample size

The CHIPS Trial will be recruiting 60 participants as dictated by sample size calculations [31]. For this pilot sub-study, a formal sample size calculation has not been performed. Instead, a pragmatic approach dictated by resource and personnel constraints has been adopted and up to 20 participants will be enrolled. All participants will consent to participate in the sub-study at the time of trial enrolment and will provide verbal consent to participation at the first time point (24 h post-challenge).

### Study procedures

**1. The capture of environmental Strep A in the vicinity of a potential infection**

Experiments will be completed at three timepoints: 24, 36 and 48 hours following inoculation of the participant with Strep A (the challenge, Day 1). Prior to inoculation, one removable adhesive shelf will be placed on a solid wall 2 metres above the floor in each of the participant’s inpatient cubicles. At 09 : 00 on Day 2 (24 hours post-challenge) one horse blood agar containing colistin and nalidixic acid (HBA-CNA, Pathwest, WA, Australia) selective settle plate will be placed on each shelf and another on the overbed tables of participants. HBA-CNA settle plates that allow Strep A growth and minimize the overgrowth of swarming Gram-negative bacteria will be used throughout [34]. These will remain in position for 4 hours and be removed at 13 : 00. This same process will be repeated on Day 3 (48 hours post-challenge). Settle plates will also be placed in the same positions described above whilst participants are sleeping (approximately 36 hours post-challenge). These will be placed at 22 : 00 on Day 2 (~36 hours post-challenge) and remain in place for 8 hours, before removal at 06 : 00 on Day 3.

**2. The distance that Strep A droplets move beyond a suspected infection**

**Fig. 1.**
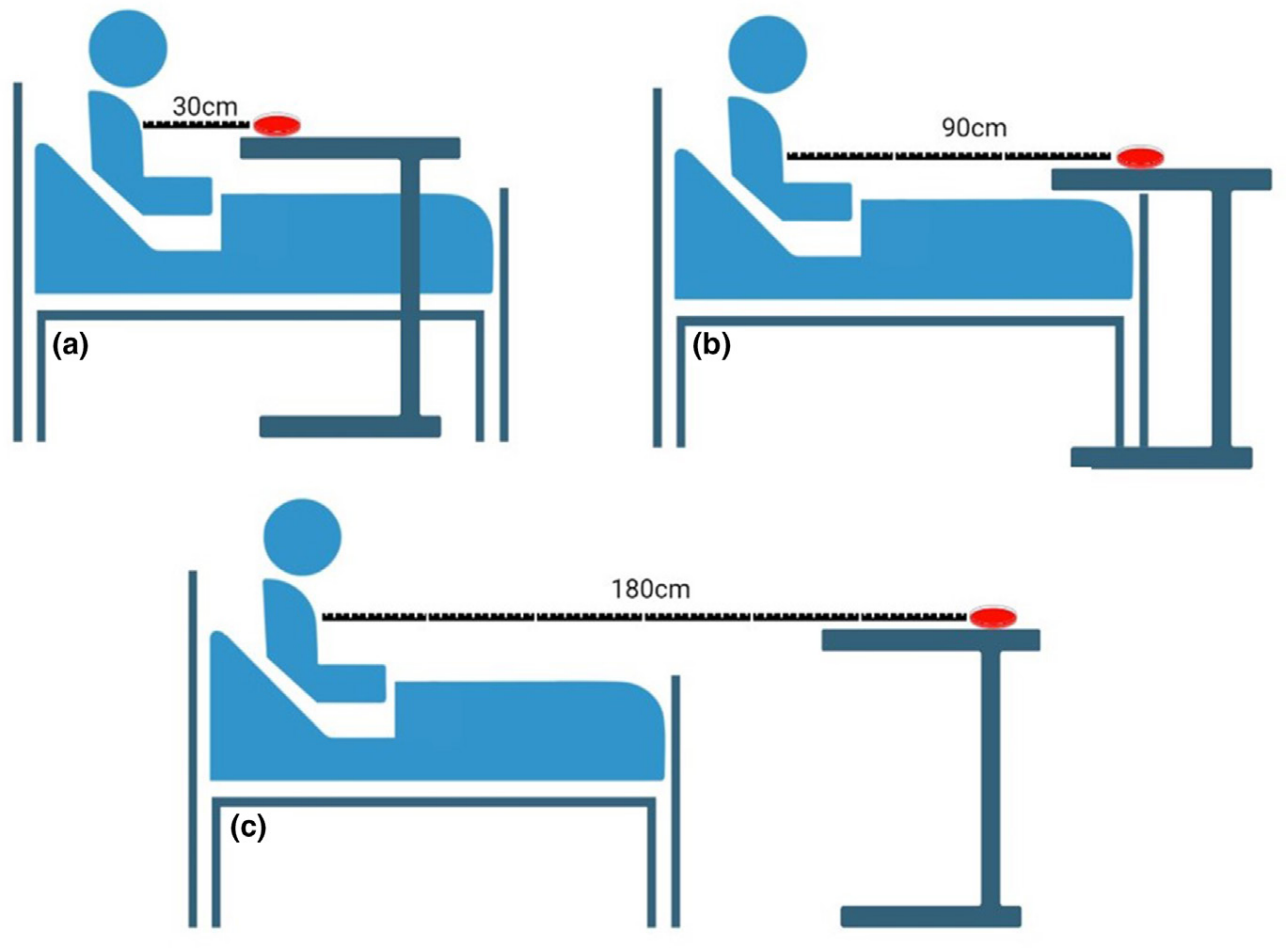
A graphical depiction of transmission experimentation with agar plates placed (**a**) 30 cm, (**b**) 90 cm and (**c**) 180 cm from the chest of the participant as they count from 1 to 100 for one minute.

These experiments will be completed at two time points: 24 hours post-challenge and 48 hours post-challenge, commencing at 09 : 00 on Days 2 and 3, respectively. Participants will be seated on their beds in front of a table draped with a sterile mat and adjusted to be the same height as their chest. The table will be exactly 30 centimetres (cm) from the chest of the participant and will hold the HBA-CNA settle plate (Fig. 1). To understand transmission during speaking, the participant will be asked to count upwards from one in a conversational tone for one minute as recorded by a stopwatch. At the conclusion of the minute, the participant will cease counting, and the plate will remain in place for an additional minute to capture any droplets that may still be falling. The experiment will be repeated in the manner described above with new plates placed at distances of 90 and 180cm [14, 28].

**3. The environmental assessment of the confinement room via swabbing**

**Fig. 2.**
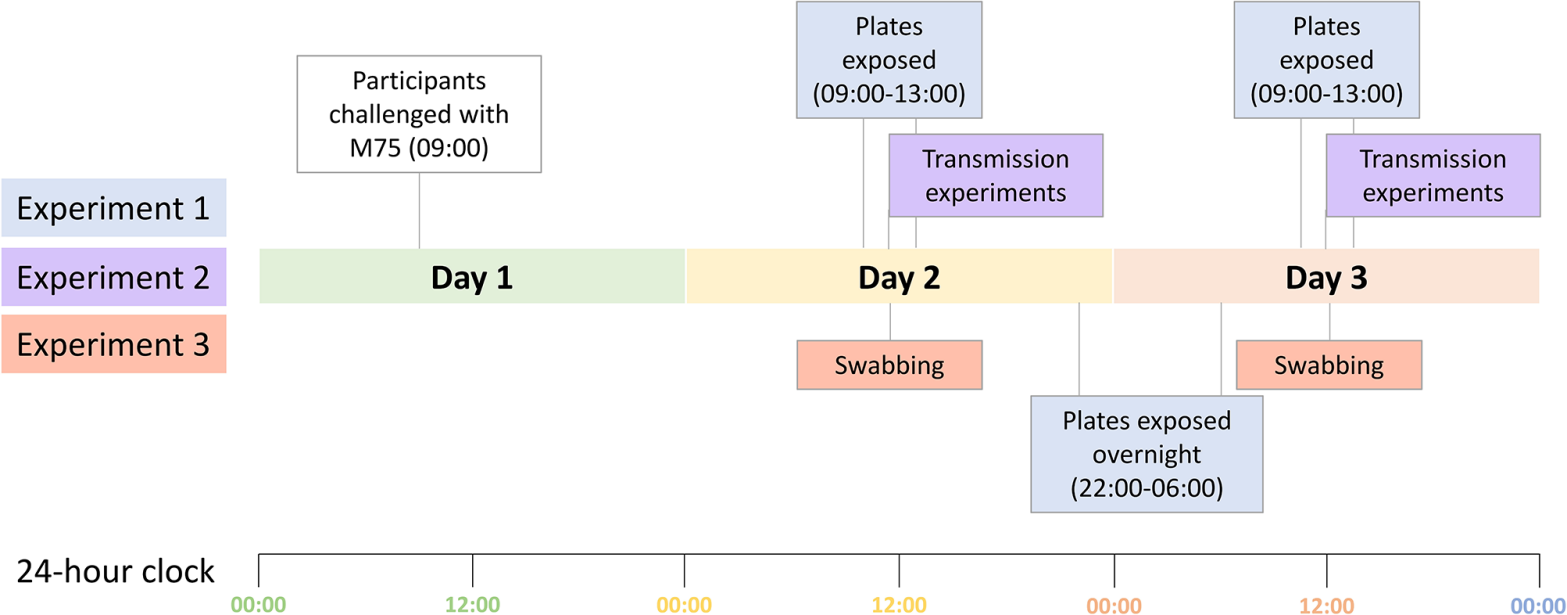
A timeline of experimentation over days 1–3 of the CHIPS trial.

Following the completion of the droplet transmission experiments, environmental swabs of five items belonging to each participant will be collected. These will include one personal item as determined by the participant, the bed remote control, bedside table, water bottle/cup and intravenous (IV) stand. Flocked swabs (Conan regular flocked swab breakpoint in peel pouch sachet; Copan, Italy) selected for appropriateness of use and transport with our selected medium will be moistened with two to three drops of sterile saline and rolled over the selected object/surface in at least two different diagonal directions across a surface area of approximately 25 cm^2^. Swabs will be placed immediately in a cryovial containing 0.5 ml of skim milk, glucose, glycerol broth (SGGB) solution (PathWest Media) kept at 4–8 °C. In addition, 10 common, high-touch areas in the confinement room will be swabbed using the same methodology, selected based on observation of the room during morning vital checks. A timeline demonstrating when each experiment will occur can be seen in Fig. 2.

### Transport of materials

Upon removing each HBA-CNA settle plate from their location, lids will be replaced, secured with tape and placed upright in a sterile bag. All SGGB cryovials will remain upright in a specimen transport container. All samples will be placed in an onsite refrigerator (4–8 °C) until ready for transport to the laboratory in an esky containing ice bricks, to maintain the transportation temperature below 10 °C. All samples will be transported within 8 hours of collection to the laboratory, with no samples remaining in the esky longer than 45 minutes.

### Microbiological analysis

All swabs and CNA plates collected will undergo transfer and processing for microbial culture for beta-haemolytic streptococci (BHS) using gold standard culture methodology [35] and according to Clinical and Laboratory Standards Institute (CLSI) standards. If no bacterial growth is observed after 24 hours, incubation will be extended for a further 24 hours to allow growth of slow-growing or small colonies. The presence of Strep A as indicated by β-haemolytic morphology will be confirmed with subculture, bacitracin sensitivity testing and positive group A latex agglutination reaction (Streptex, Thermo Scientific). Strep A isolates will be stored at −80 °C to permit further strain characterization. Results will be reported as the presence or absence of Strep A on each sample. Quantitation of the amount of Strep A is not possible with this experimental design.

### Statistical analysis

Investigators will remain blinded to the penicillin dosage received by each participant until all data are collected. Each binary outcome (presence or absence of Strep A on collected samples) will be assessed against the dose received by participants and whether they were confirmed as meeting the primary study endpoint (diagnosed pharyngitis). Such analysis will allow for an assessment of transmission potential among symptomatic, asymptomatic and non-cases. Frequencies and percentages will be summarized and chi-square statistics will be used for further analyses.

### Ethics and dissemination

This sub-study is included in the CHIPS Trial protocol, which has been reviewed and approved by Bellberry Human Research Ethics Committee (approval 2021-03-295). The CHIPS Trial is registered with the Australian New Zealand Clinical Trials Registry (ACTRN12621000751875). Findings will be presented at national/international forums and reported in peer-reviewed publications.

## Discussion

Early studies investigating the transmission of Strep A in the 1950s by Hamburger *et al*. [14, 15, 36, 37] enhanced scientific understanding of how to prevent human-to-human transmission and have been relied upon to this day – including informing the methodology of this sub-study. Notably, Hamburger and colleagues identified Strep A to have transmission potential of up to 9.5 feet (2.9 metres) in those with a symptomatic infection whilst sneezing, although very limited transmission potential was identified at any distance whilst talking [14]. The authors believe there is a benefit in contemporary replication to confirm these findings while ascertaining whether airborne or other methods of transmission may also occur. These proposed experiments capitalize on a human challenge study being conducted and provide the opportunity to increase our understanding of Strep A transmission mechanisms in the modern era.

The timing of experimentation, specifically settle plate placement and swabbing, coincides with the periods of maximal movement in the room as participants are assessed for potential symptoms of pharyngitis and have samples taken. Hence it is expected that this will be the period where transmission potential is at its highest – a study strength. While practice with HBA-CNA plates suggests exposure for no more than 4 hours at room temperature to avoid excessive contamination and degradation of the agar [38], we hypothesize that reduced movement in the room and lower room temperatures overnight will be conducive to extending exposure of these plates to 8 hours without a cover.

Lack of facilities and resources that enable strict infection control measures (such as being able to isolate participants in a single room including own bathrooms, etc.) has often been identified as a barrier to undertaking human infection/challenge studies [39]. In our study, participants are not in separate rooms but in a hospital ward-style beds, divided by curtains. One incidental benefit of our experiments would be to demonstrate whether standard infection control measures alone (without physical isolation) are adequate for prevention of Strep A transmission and thereby demonstrate the safety of similar research studies. The penicillin concentration received by the participant will need to be factored into these observations, as current practice is to deisolate those infected with Strep A after 24 hours of appropriate antimicrobial therapy.

This above point is, however, a limitation of the methodology, with contamination a possibility. Individual participants are the only ones with access to their belongings swabbed in experiment 3, so the authors believe there to be limited chance of cross-contamination between participants. As participants keep their curtains closed droplet contamination is unlikely on any plates, but potential airborne spread remains a possibility. This is also possible with the overbed plates placed at a height of 2 metres. As all participants are being given the *emm75* strain of Strep A, the only way to confirm contamination is if the samples of a participant are positive for Strep A despite no nasal or throat samples being taken from the participant confirming this. Symptomatic assessment will also be referred to should this occur.

These experiments have several limitations. Firstly, it is a small pilot study of only 20 participants due to resource and personnel constraints. However, this is the first time similar experiments have been conducted to improve our knowledge of Strep A transmission in more than 70 years. Participants receive a placebo or varying concentrations of penicillin infusion, which may reduce the likelihood of Strep A inoculating the agar settle plates or surface swabs. This limitation is overcome by inclusion of a placebo and all participants being inoculated with the same standardized dose of *emm*75. There is a possible limitation in restricting the distances in experiment 2 to 180 cm; however limitations of space in the confinement facility prevented further extension of this experiment without potentially exposing staff and other participants. Further, the CRO deemed asking challenged participants to cough or sneeze at these distances to be an unacceptable level of exposure risk to others; thus, talking was selected as an appropriate intermediary. Finally, time and resource constraints have limited the use of more specialized equipment – such as microbial air samplers – to determine the influence of factors such as airflow and air exchange rate [40] in this study, although the research team are presently investigating the possibility of employing such methods in households where the burden of Strep A is high. The results from this sub-study will of necessity precede the development of future protocols.

In conclusion, any results elicited from these experiments will be of benefit to the scientific literature in improving our knowledge of opportunities to prevent Strep A transmission as a direct component of the primordial prevention of rheumatic fever. Acute rheumatic fever remains an uncontrolled risk in low- and middle-income settings, and impoverished populations in high-income settings. New strategies for all levels of prevention are needed. Our work provides an opportunity to make important advances towards better understanding of opportunities for prevention.
